# Prevalence of coronary atherosclerotic plaque in patients with a low coronary artery calcium score

**DOI:** 10.1186/1753-6561-6-S4-P25

**Published:** 2012-07-09

**Authors:** VS Mehta, M Patel, S Venuraju, A Jeevarethinam, A Yerramasu, A Lahiri

**Affiliations:** 1 University College London, UK; 2 Clinical Imaging and Research Centre Wellington Hospital, London, UK; 3 Imperial College, London, UK; 4 Middlesex University, London, UK

## Introduction

Patients with low coronary artery calcium (CAC) scores are deemed to be at low risk of future cardiac events. It is unclear what the prevalence of significant coronary disease is in these patients. It is also not established what cluster of traditional cardiovascular risk factors predict the presence of any coronary plaque in this group. We hypothesised patients with a calcium score of < 10 Agatston Units may have significant coronary plaque lesions that would be picked up on a more robust method for the evaluation of coronary plaques such as CT coronary angiography (CTCA).

## Methods

We evaluated 96 patients with coronary artery calcium score < 10 Au referred for CTCA with suspected coronary disease. Plaques causing > 50% stenosis on CTCA were deemed to be significant. Univariate and multivariate analysis using logistic regression was carried out to evaluate which traditional risk factors predicted the presence of plaque in this cohort. A p-value<0.05 was considered significant.

## Results

Of the 96 patients included in the study, a mean age of 52.7+11.4 and 62.5% were male. Presence of any coronary artery plaque was noted in 19 (19.8%) of patients and 7 (7.3%) of patients had at least one plaque lesion causing > 50% stenosis. In patients with zero calcium score only, 11 (13.9%) and 3 (3.8%) patients were found to have any coronary plaque and at least one plaque causing >50% stenosis. In a univariate analysis of the entire cohort, only age (p=0.02), presence of any calcium (p=0.01), and family history of significant CAD (p=0.037) were found as predictors of plaque, whereas in a multivariate analysis, only age and calcium score retained significance (p=0.05 and 0.035).

## Conclusions

Patients with a low CAC score (<10 Au) have a low but not insignificant prevalence of >50% plaque and a full angiographic evaluation of the coronary arteries is essential in older patients and those with a strong family history of CAD. Our study was not powered to predict the effect of risk factors on the prevalence of significant plaque in the entire cohort as well as in the sub-group of patients with zero calcium.

